# Microscope-Assisted Excision of CT-Occult Inferior Rectus Adhesions After Pediatric Orbital Floor Trapdoor Fracture Repair: A Case Report

**DOI:** 10.7759/cureus.116025

**Published:** 2026-09-09

**Authors:** Akira Ishida, Tomoyuki Kashima

**Affiliations:** 1 Oculoplastic Surgery, Oculofacial Clinic Group, Tokyo, JPN

**Keywords:** ct-occult adhesions, forced duction test, inferior rectus muscle, microscopic scar excision, pediatric orbital floor fracture, persistent diplopia, revision orbital surgery, trapdoor fracture

## Abstract

We report the case of a 12-year-old competitive athlete who presented with persistent ocular motility restriction and diplopia following surgical repair of an orbital floor trapdoor fracture. Although the initial surgery was performed at another institution on the day following the injury and postoperative CT confirmed the successful release of tissue entrapment, her clinical symptoms persisted (elevation restriction grade -3). Revision orbital surgery was performed 53 days after the initial injury. Pre-incision forced duction testing (FDT) confirmed mechanical tethering. Under microscopic guidance, meticulous adhesiolysis and scar excision were conducted to completely remove dense fibrotic-appearing adhesions on the orbital floor and the inferior surface of the inferior rectus muscle. Intraoperative FDT immediately converted to negative. Six months postoperatively, ocular motility completely resolved to grade 0, and the threshold for diplopia on the binocular single vision field improved substantially from 25° to 40°, allowing her to return to competitive sports without functional impairment. The meticulous observation and excision of these CT-occult scar-like adhesions using an operating microscope contributed significantly to the successful functional recovery. This case demonstrates that microscopic scar excision can be an effective intervention for persistent motility restriction after trapdoor fracture repair, even when residual entrapment has been resolved on CT.

## Introduction

Pediatric orbital floor fractures increase in incidence after the age of seven, coinciding with the development of the midface and paranasal sinuses [1,2]. A distinguishing feature of these injuries in children, compared to adults, is the high prevalence of trapdoor-type fractures [3-5], which frequently occur during sports-related activities [5]. Due to the greater elasticity of the pediatric skeleton, the fractured bone segment often snaps back into its original position like a hinge, a phenomenon known as the trapdoor mechanism, entrapping the inferior rectus (IR) muscle and surrounding periorbital soft tissues [1-6]. This mechanism often triggers the oculocardiac reflex, a clinical triad of bradycardia, nausea, and syncope resulting from traction on extraocular muscles, leading to clinical manifestations such as diplopia, ocular motility restriction, and systemic symptoms like nausea [7-9].

To prevent tissue ischemia and resultant irreversible fibrosis, surgical intervention within 24 to 48 hours is widely recommended [1,2,4,7,8]. A shorter interval between injury and surgery is associated with lower rates of residual diplopia. Early intervention within 48 hours typically results in residual symptoms in only 0-15% of cases, whereas delayed treatment increases this risk significantly to 30-60% [8,10,11]. However, a diagnostic paradox often arises where clinical symptoms persist even when postoperative computed tomography (CT) confirms successful anatomical release. Mechanically, the entrapped or scarred IR muscle acts as a "leash" on the globe; while the IR is primarily a depressor, its inability to elongate due to scarring prevents the eyeball from rotating superiorly. This results in vertical ocular misalignment during upward gaze, causing vertical diplopia that severely compromises stereopsis (high-grade binocular depth perception), especially in high-demand patients such as athletes.

However, there are currently no definitive guidelines or consensus for managing patients who suffer from persistent diplopia and motility restriction despite receiving timely and technically appropriate primary surgery. While some cases may improve with conservative observation, others show no progress, and the optimal timing or surgical technique for revision surgery remains controversial [1,6,9-12]. To our knowledge, microscope-assisted excision of CT-occult peri-IR scar tissue in a pediatric trapdoor fracture, combined with immediate intraoperative normalization of forced duction, has not been specifically described.

In this report, we present the case of a top-level competitive young athlete who experienced persistent ocular motility restriction after trapdoor fracture repair, despite postoperative CT confirming the successful release of tissue entrapment. This case highlights that fibrotic-appearing adhesions involving the IR muscle can cause persistent motility dysfunction even in the absence of CT-detectable entrapment. Our findings suggest that adhesiolysis and scar excision using an operating microscope to enhance surgical precision can be effective interventions for achieving functional recovery in these challenging cases.

## Case presentation

A 12-year-old girl presented with persistent diplopia during upward gaze following an orbital floor trapdoor fracture of the right eye caused by a sports-related injury (Figure 1A). Although she had undergone emergency reconstructive surgery at another hospital on the day following the injury, her symptoms did not resolve. She was referred to our department 40 days after the initial surgery. The patient complained of diplopia that became more prominent with fatigue, though she denied any pain on gaze. To identify the cause of the persistent restriction, a structured diagnostic approach was employed. Third nerve palsy was considered unlikely because the restriction was isolated to elevation, with preserved horizontal and downward motility and no ptosis or pupillary abnormality. Traumatic myositis was also considered less likely because there was no pain on gaze, conjunctival injection, or significant intramuscular edema on CT. Postoperative CT revealed an orbital floor defect measuring approximately 10 mm in the anteroposterior diameter, with no obvious mechanical interference with the IR muscle by the inserted plate (Figure 1B). The IR muscle maintained a normal anatomical position and continuous contour without any clear signs of pathologic rounding, significant intramuscular edema, or structural tissue entrapment. Clinical examination revealed a marked limitation of upward gaze in the right eye, quantified as a -3 restriction on a scale of 0 (normal) to -4 (no movement beyond the midline) (Figure 2A). A Hess chart confirmed severe restriction of elevation (Figure 2B), and binocular single vision field (BSVF) testing demonstrated diplopia at an upward gaze of 25° or more (Figure 2C).

**Figure 1:**
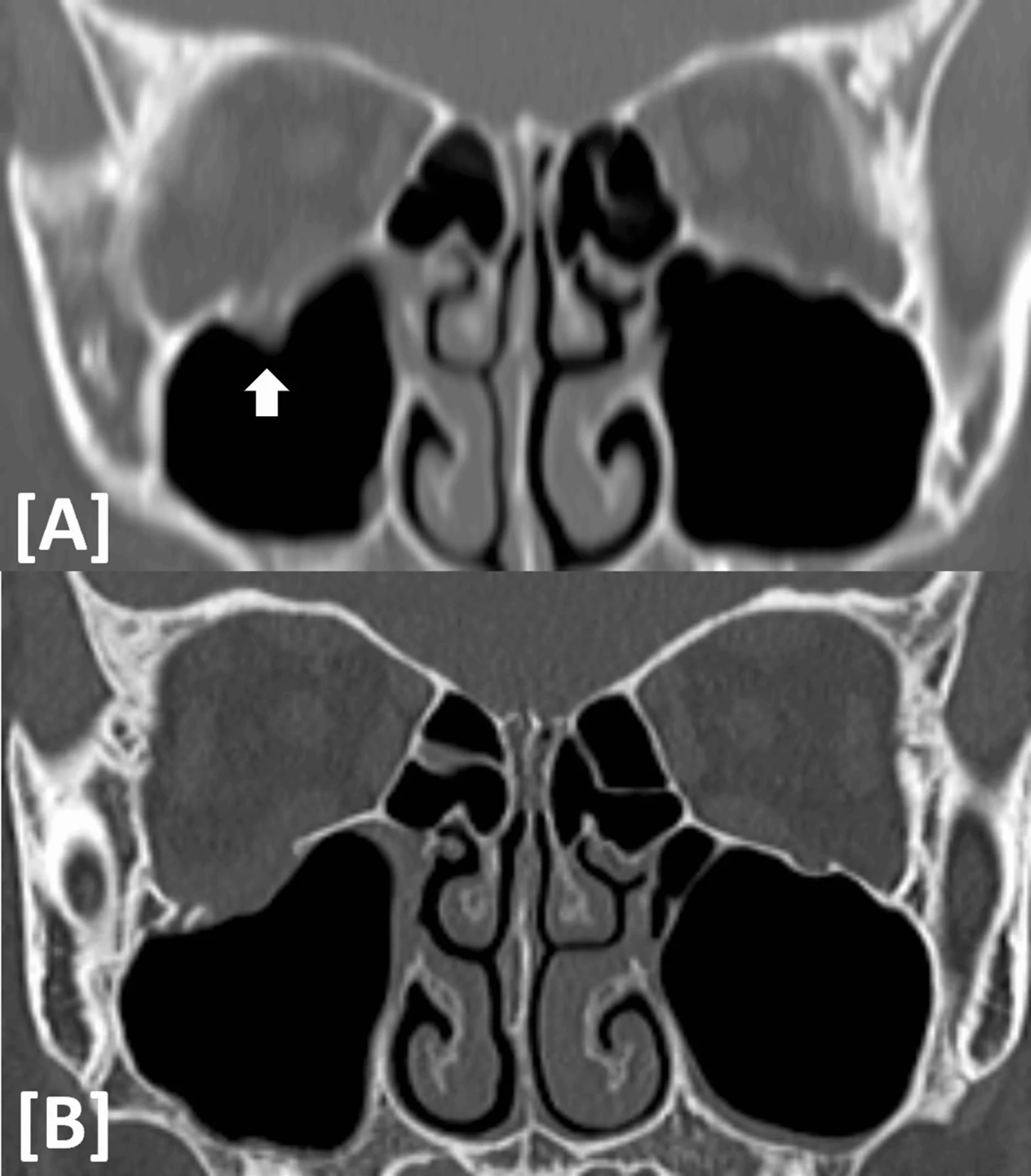
Preoperative and postoperative computed tomography (CT) scans from the primary surgery. (A) Initial CT scan obtained at the previous hospital prior to the primary surgery, showing a right orbital floor trapdoor fracture with soft tissue entrapment (indicated by the white arrow). (Reproduced with permission from the referring institution). (B) Follow-up CT scan at the initial visit to our hospital (after the primary surgery), demonstrating that the tissue entrapment has been resolved on CT.

**Figure 2:**
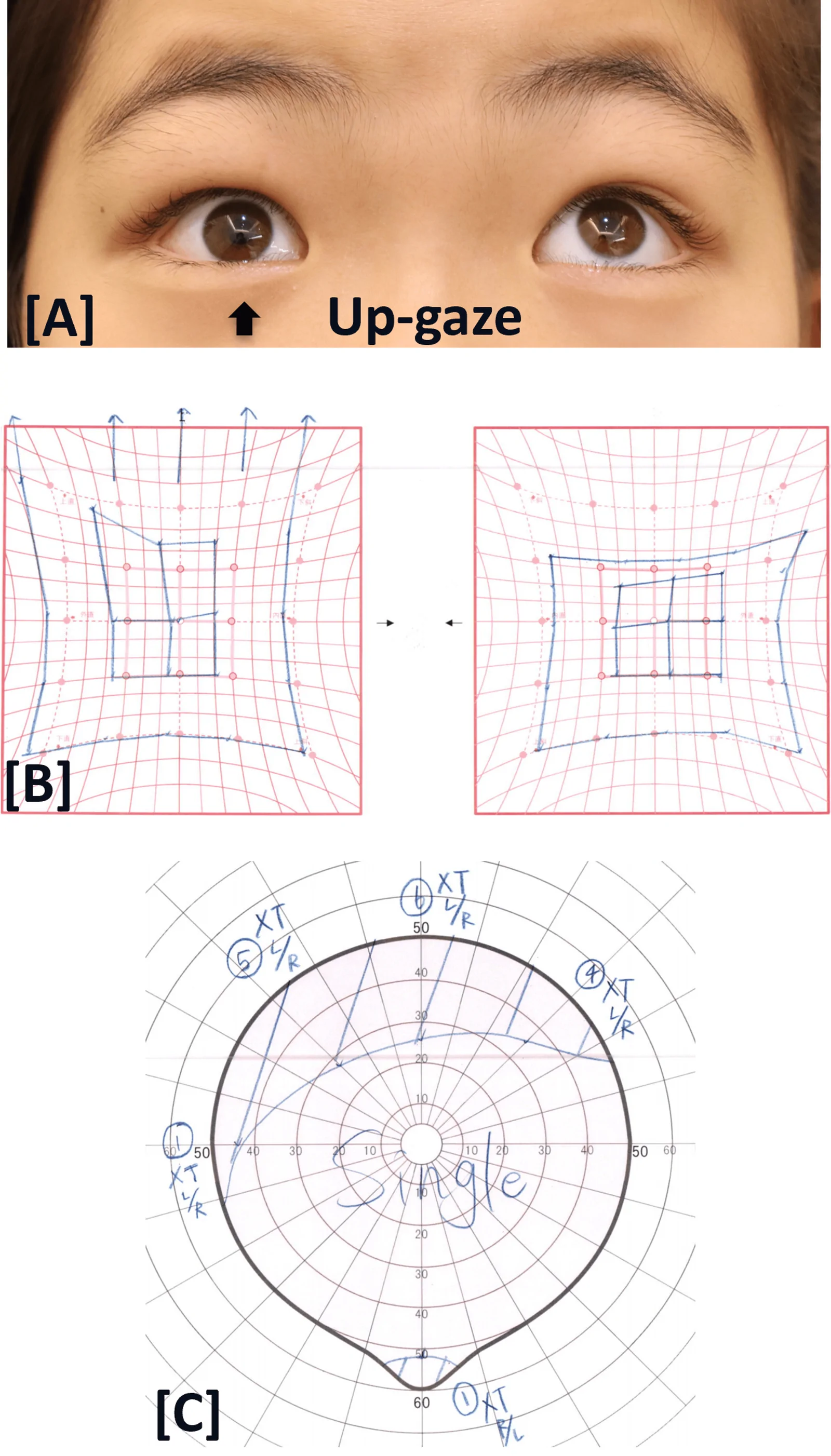
Baseline clinical and functional evaluations at our department (40 days post-primary surgery). (A) Ocular motility photograph showing marked limitation of upward gaze in the right eye, quantified as a -3 restriction (gaze direction is indicated by the black arrow). (B) Pre-revision Hess chart showing pronounced restriction of elevation in the right eye. (C) Pre-revision BSVF testing showing diplopia present at an upward gaze of 25° or more. Note for non-specialists: On the Hess chart, the grids represent the field of vision; a smaller or distorted grid indicates muscle underaction or restriction. In the BSVF testing, the non-shaded regions represent areas of binocular single vision, while the shaded areas indicate where diplopia occurs. Up-gaze, upward gaze. (B) and (C) Photographic/scanned records of the original clinical test printouts, with the patient examination tracings appearing in the standard colors generated by the testing apparatus. BSVF: Binocular single vision field

Due to the patient’s strong desire to return to high-level competitive sports, a revision surgery was performed 53 days after the initial injury using an operating microscope. Prior to the surgical incision, a forced duction test (FDT) was performed under general anesthesia to avoid patient discomfort and eliminate potential interference from active muscle contraction. The FDT was positive, confirming profound mechanical tethering of the globe. Intraoperatively, although the previous entrapment of orbital tissue had been resolved, dense fibrotic-appearing adhesions were observed. These had formed between the capsule of the bioabsorbable plate (composed of unsintered hydroxyapatite/poly-L-lactic acid (u-HA/PLLA)), the periosteum posterior to the plate, the IR and inferior oblique muscles, and the surrounding orbital soft tissues. Notably, dense adhesions were found on the inferior surface of the IR muscle (Figure 3A). It was determined that these dense scar tissues directly inhibited the gliding of the IR muscle, serving as the primary cause of the restricted upward gaze.

**Figure 3:**
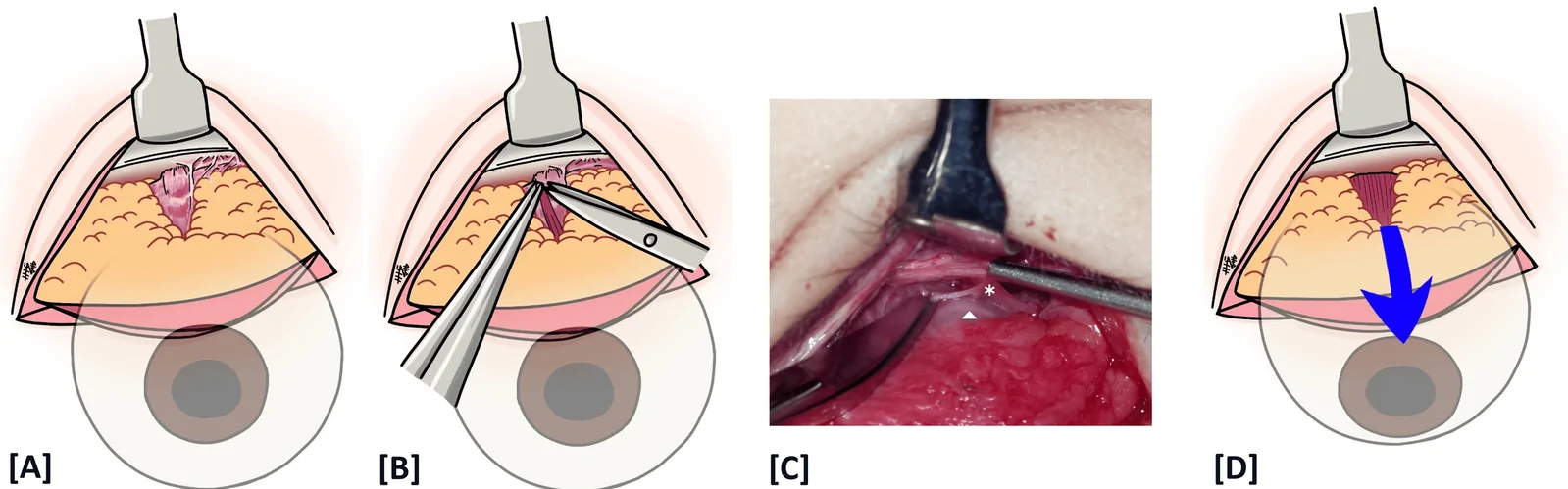
Intraoperative microscopic schematics and photograph of the revision surgery. (A) Schematic illustration showing the fibrotic-appearing adhesions formed between the orbital floor (including the u-HA/PLLA plate capsule) and the IR muscle. (B) Schematic illustration demonstrating meticulous microscopic excision of the scar tissue. (C) Intraoperative microscopic photograph showing dense scar-like fibrous tissue (asterisks) formed on the inferior surface of the IR muscle (arrowheads). (D) Schematic illustration showing complete release of the IR muscle and restored tissue gliding after thorough removal of the scarring. Source: Original schematics in A, B, and D commissioned by the authors, created by Dr. Yutaka Nakamura using CLIP STUDIO PAINT PRO (version 5.1.1; Celsys, Inc., Tokyo, Japan) on an iPad Pro (4th generation) with Apple Pencil (Apple Inc., Cupertino, CA, USA). Used with permission. IR: Inferior rectus

Under microscopic guidance, meticulous adhesiolysis of the focal yet dense scar tissue was performed with high precision; the scar tissue was not merely separated but thoroughly excised (Figures 3B, 3C). Thin layers of scarring along the IR surface were also removed. Immediately following the total excision of this dense scar plate, the intraoperative FDT was repeated and confirmed to have converted to completely negative, indicating immediate relief of the mechanical restriction (Figure 3D). Inspection of the orbital floor following total scar excision revealed adequate structural stability of the small bony defect without significant residual gap; therefore, the implant was completely removed and no new implant was placed. At the conclusion of the surgery, 20 mg of triamcinolone acetonide was administered into the intraorbital space with the aim of reducing postoperative inflammation and the risk of re-adhesion. No specific restrictions were placed on postoperative ocular motility; instead, the patient was instructed to perform active eye movements to promote postoperative ocular excursion and potentially reduce re-adhesion. CT one week postoperatively confirmed plate removal without residual soft tissue entrapment or new bony abnormality (Figure 4A).

**Figure 4:**
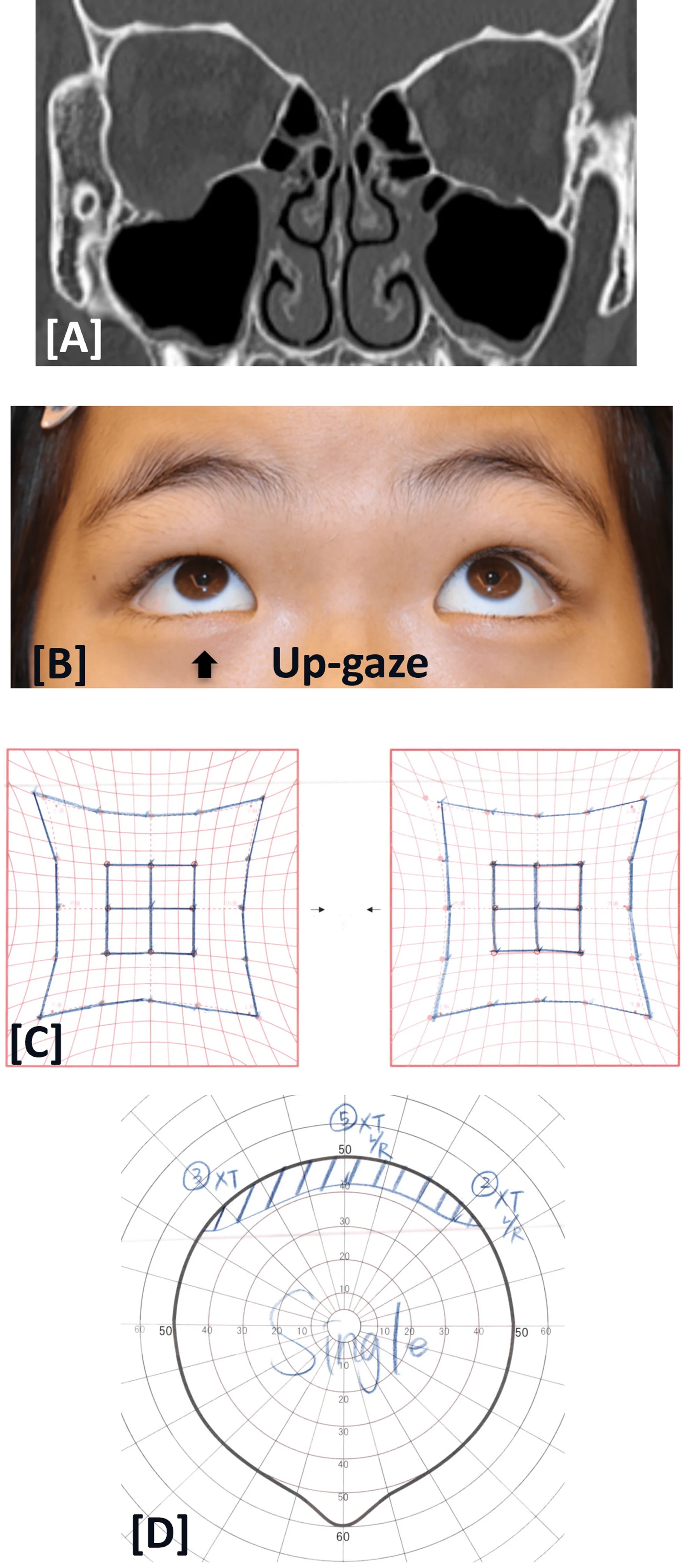
Postoperative clinical course and functional recovery after revision surgery. (A) Post-revision CT showing removal of the implant without residual tissue entrapment or new bony abnormality. The fibrotic-appearing adhesions identified intraoperatively were CT-occult (not discernible on the pre-revision CT). (B) Ocular motility photograph at the six-month follow-up, demonstrating marked improvement in ocular motility, with complete restoration of elevation to grade 0. (C) Post-revision Hess chart at six months, demonstrating recovery to within the normal range. (D) Post-revision BSVF testing at six months showing substantial expansion of the single vision field, with residual diplopia limited to extreme upward gaze of 40° or more. (C) and (D) Photographic/scanned records of the original clinical test printouts, with the patient examination tracings appearing in the standard colors generated by the testing apparatus. BSVF: Binocular single vision field; up-gaze: upward gaze

The postoperative course was favorable. By three months, the patient’s subjective diplopia had improved to the point where it no longer interfered with her performance in competitive sports. At the six-month follow-up, the upward gaze restriction completely resolved to a grade of 0 (normal) (Figure 4B). Hess chart testing demonstrated recovery to within the normal range (Figure 4C). Concurrently, BSVF testing showed that the single vision field substantially expanded, with the diplopia threshold improving from 25° to 40° or more, demonstrating functional recovery that allowed full return to high-level competitive sports without any residual prism or compensatory head posture (Figure 4D). The overall clinical course and key milestones of the patient are summarized in Table 1.

**Table 1:** Summary of the clinical course and key milestones.

Time Point	Clinical Event/Status	Key Findings/Interventions
Day 0	Initial Injury	Sports-related right orbital floor trapdoor fracture
Day 1	Primary Surgery	Emergency reconstructive surgery (at another institution)
Day 40	Initial Visit (Referral)	Persistent vertical diplopia in upward gaze with no residual entrapment on CT
Day 53	Revision Surgery	Microscopic adhesiolysis and total scar excision
3 Months Post-op	Follow-up	Subjective resolution of diplopia; return to competitive sports
6 Months Post-op	Final Follow-up	Near-complete functional resolution; residual diplopia strictly limited to extreme upward gaze (≥40°)

## Discussion

In pediatric orbital floor trapdoor fractures, persistent postoperative ocular motility restriction may arise from transient factors such as surgical edema, hemorrhage, or temporary nerve palsy, which typically improve with observation. However, if the restriction is caused by residual entrapment or adhesions between orbital tissues, fractured bone fragments, and the implant, conservative management carries a significant risk of irreversible muscle fibrosis, leading to permanent functional impairment [8,11,13-15]. Residual diplopia necessitating secondary strabismus surgery often results in a prolonged and complex treatment course [16]. The IR muscle is particularly susceptible to fibrosis following surgical manipulation, frequently becoming the primary source of motility dysfunction [4,13,14,17].

While CT remains the gold standard for evaluating bony structures, it lacks the soft-tissue resolution required to identify fine fibrotic bands or subtle soft-tissue changes [1,13]. Although no evidence-based threshold for revision surgery has been established, persistent severe motility restriction beyond several weeks, particularly when accompanied by a positive forced duction test and no residual entrapment on CT, should raise suspicion for CT-occult fibrotic-appearing adhesions rather than transient postoperative edema alone. While magnetic resonance imaging (MRI) may provide complementary visualization of extraocular muscle and perimuscular soft-tissue changes when CT findings are inconclusive, whether thin, focal postoperative adhesions such as those observed in the present case can be reliably identified by MRI remains uncertain. In the present case, the patient was an elite-level youth athlete whose performance would be directly compromised by any degree of diplopia within her visual field. Therefore, we proactively opted for revision surgery to achieve maximal functional recovery, which ultimately led to a successful outcome.

Our intraoperative findings confirmed that the original tissue entrapment had been released. However, dense fibrotic-appearing adhesions involved the plate capsule, posterior periosteum, IR and inferior oblique muscles, and surrounding orbital soft tissues. Adhesions along the inferior surface of the IR muscle appeared to directly impair muscle gliding and were considered the principal cause of the persistent elevation restriction. Furthermore, no obvious degeneration of the IR muscle was observed. These findings indicated that dense scar tissue between the orbital floor and the IR muscle was the primary cause of the upward gaze limitation. Recent evidence has further demonstrated that IR adherence to fibrous tissue surrounding orbital implants can itself produce severe postoperative motility restriction [18]. In contrast to cases where post-repair elevation restriction stems primarily from muscular swelling and resolves with conservative observation [19], our patient showed neither significant muscular edema on CT nor spontaneous recovery over several weeks. The pre-incision FDT under general anesthesia was positive, confirming profound mechanical tethering. Continued observation might have allowed further maturation of the adhesions; however, the natural history of this condition remains uncertain. Given the persistent severe restriction, positive FDT, and the patient’s high functional demands, revision surgery was selected after careful discussion with the patient and her family.

Regarding the surgical technique, we determined that simple adhesiolysis (releasing the adhesions) might be insufficient to prevent recurrence. Therefore, we performed a thorough excision and removal of the dense scar tissue itself. A key highlight of this procedure was the use of an operating microscope. Although the microscope is not routinely employed in orbital floor fracture repairs, microscopic magnification may improve visualization of subtle scar tissue bands and facilitate precise dissection around the extraocular muscles. This may facilitate precise release of mechanically restrictive scar tissue while minimizing unnecessary manipulation of adjacent structures. Microscopic guidance can be valuable in selected cases for the meticulous excision of such scarring. Following the complete excision, the intraoperative FDT immediately converted to negative, and 20 mg of triamcinolone acetonide was administered intraorbitally with the aim of reducing postoperative inflammation and the risk of re-adhesion.

While conventional secondary strabismus surgery (e.g., muscle recession) aims to rebalance muscle tension for alignment, our approach of microscope-assisted direct adhesiolysis addresses the underlying pathology by removing the CT-occult scar tissue, thereby restoring the anatomical gliding mechanism while preserving healthy extraocular muscle dynamics.

This report illustrates that microscopic intraorbital scar tissue excision can be an effective intervention for ocular motility restriction caused by scarring following orbital floor trapdoor fracture repair. This case suggests that in pediatric patients with persistent motility restriction after initial repair, even in the absence of residual entrapment on CT, the possibility of fibrotic-appearing adhesions should be considered, and microscopic adhesiolysis may be a valuable option for achieving successful functional recovery.

This report has several limitations. First, histopathologic confirmation of the excised tissue was not available; however, the gross intraoperative appearance was consistent with dense scar tissue. Second, because implant removal, microscopic adhesiolysis and scar excision, intraorbital triamcinolone administration, and postoperative ocular motility exercises were performed as components of the same intervention, the relative contribution of each component to the long-term functional recovery cannot be determined. Nevertheless, the immediate intraoperative conversion of the FDT to negative strongly supports the direct mechanical efficacy of the microscopic scar excision.

## Conclusions

This case illustrates that CT-occult fibrotic-appearing adhesions involving the IR muscle should be considered in pediatric patients with persistent mechanical elevation restriction after apparently successful trapdoor fracture repair. In selected cases, microscope-assisted adhesiolysis and excision of visible scar tissue may facilitate precise mechanical release and functional recovery. Further clinical experience is required to determine the optimal timing, indications, and reproducibility of this approach.
